# Biosynthesis of the Fungal Cyclic Lipodepsipeptide Pleosporacin, a New Selective Inhibitor of the Phytopathogen *Botrytis cinerea*

**DOI:** 10.1002/cbic.202500315

**Published:** 2025-05-23

**Authors:** Carsten Wieder, Rainer Wiechert, Alexander Yemelin, Louis Pergaud Sandjo, Eckhard Thines, Till Opatz, Anja Schüffler

**Affiliations:** ^1^ Institute of Molecular Physiology Johannes Gutenberg‐University Hanns‐Dieter‐Hüsch Weg 17 55128 Mainz Germany; ^2^ Institut für Biotechnologie und Wirkstoff‐Forschung gGmbH Hanns‐Dieter‐Hüsch Weg 17 55128 Mainz Germany; ^3^ Department of Chemistry Johannes Gutenberg‐University Duesbergweg 10–14 55128 Mainz Germany; ^4^ Department of Chemistry Universidade Federal de Santa Catarina Florianópolis Santa Catarina 88040‐900 Brazil

**Keywords:** biological activity, biosynthesis, cyclic lipodepsipeptides, heterologous expression, natural products, nonribosomal peptide synthetases

## Abstract

Bioactivity‐guided isolation led to the identification of the cyclic lipodepsipeptide pleosporacin (**1**) from the mycelia extract of fungal strain *Pleosporales* sp. IBWF 020‐21, a potent selective inhibitor of the fungal phytopathogen *Botrytis cinerea*. The structure and stereochemistry of **1** were elucidated by NMR and Marfey analysis, respectively. Genome mining identified a candidate biosynthetic gene cluster encoding a hexamodular nonribosomal peptide synthetases, PleA, a fatty acyl‐AMP ligase, PleB, and an aspartate decarboxylase, PleC. Reconstitution of *pleABC* allowed for heterologous production of **1** in *Aspergillus oryzae* and confirmed the identity of the *ple* cluster. Based on these findings a biosynthetic route is proposed, with PleB catalyzing lipoinitiation and PleC providing the nonproteinogenic amino acid β‐alanine for the assembly of **1**.

## Graphical abstract

Bioactivity‐guided isolation led to the discovery of a potent and selective inhibitor of the phytopathogen *Botrytis cinerea*, pleosporacin (**1**), from mycelia extracts of the fungal strain *Pleosporales* sp. IBWF 020‐21. The biosynthesis of the nonribosomal cyclic lipodepsipeptide **1** was elucidated via heterologous reconstitution in *Aspergillus oryzae*.

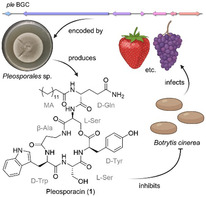

## 1 Introduction

Nonribosomal peptide synthetases (NRPS) are giant multimodular enzyme assembly lines, which facilitate the condensation of amino acids in a ribosome‐independent manner. Each module obligatory harbors a minimal set of an adenylation (A), thiolation (T), and condensation (C) domain. These are required for activation of the amino acid building blocks (A‐domain), tethering (T‐domain), and catalyzing peptide bond formation (C‐domain).^[^ ^1^ ^]^ Synthesis proceeds via repeated condensation of the amine of the T_*n*+1_‐bound amino acid with the carbonyl of the T_*n*_‐bound peptidyl chain. In contrast to ribosomal peptide synthesis, NRPS can also incorporate nonproteinogenic amino acids and hydroxy acids,^[^ ^2^ ^]^ the latter of which are esterified to yield depsipeptides.^[^ ^3^ ^]^ Optionally, modules can include tailoring domains, such as *N*‐methyltransferase domains and epimerization (E) domains that catalyze l to d stereoinversion of tethered amino acids prior to condensation. In fungal NRPSs, the release of nascent peptides is frequently catalyzed by a terminal condensation (C_T_) domain via hydrolysis, lactonization/ lactamization, or Dieckmann cyclization.^[^ ^4^ ^]^ In the special case of lipopeptides, an additional first step known as lipoinitiation is required to incorporate the fatty acyl moiety. During lipoinitiation in fungal biosynthetic pathways, a free fatty acid is activated by an acyl‐AMP ligase and subsequently transferred to an N‐terminal T_0_‐domain to prime the assembly line.^[^ ^5^, ^6^, ^7^ ^]^ Elongation of the peptidyl chain then proceeds as described. Many lipopeptides, such as echinocandins, daptomycin, and surfactin, exhibit industrially relevant antimicrobial activity and it was previously shown that the fatty acyl moiety plays an essential role therein.^[^ ^8^ ^]^ The rise of fungicide resistance in phytopathogenic fungi is a global threat to food production^[^ ^9^ ^]^ and therefore the nutrition of the steadily growing world population. This necessitates not only the discovery of new lead structures for the development of drugs but also the identification of new molecular targets or mechanisms that circumvent resistance development. Microbial natural products have been a continuous source of drug leads over the last decades.^[^ ^10^ ^]^ The discovery of selective antimicrobial agents is of particular interest for plant protection, as high selectivity reduces the impact on the environment, which is in line with the European Geen Deal (Directive 2009/128/EC).

Here, we report the bioactivity‐guided isolation, characterization, and elucidation of the biosynthesis of a new cyclic lipodepsipeptide pleosporacin (**1**), which exhibits potent selective inhibitory activity against the grey rot pathogen *Botrytis cinerea*.

## 2 Results and Discussion

**Figure 1:**
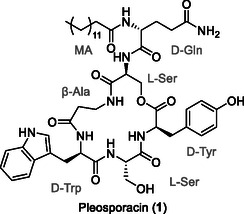
Structure of pleosporacin (**1**).

In an ongoing effort to identify new antifungal compounds, we isolated fungal strain IBWF 020‐21 (Figure S1, Supporting Information), crude extracts of which exhibit potent conidia germination inhibitory activity against the gray rot pathogen *B. cinerea*. DNA‐sequencing of the ITS fungal barcode region (Table S2, Supporting Information) frequently used for species identification^[^ ^11^ ^]^ did not suffice for species determination; however, the strain can confidently be assigned to the order *Pleosporales*. Bioactivity‐guided isolation of the active constituent led to the identification of the new cyclic lipodepsipeptide **1**, which we termed pleosporacin. The structure and stereochemistry of **1** were elucidated by NMR spectroscopy and Marfey analysis, respectively (**Figure** **1**). Compound **1** features a macrocyclic scaffold composed of myristic acid (MA), d‐Gln_1_, l‐Ser_2_, β‐Ala_3_, d‐Trp_4_, l‐Ser_5_, and d‐Tyr_6_ with the macrolactone ester bond formed between the β‐hydroxy moiety of l‐Ser_2_ and the carboxyl‐moiety of d‐Tyr_6_. Structurally, **1** is closely related to previously reported cyclic lipodepsipeptides, namely, symbiosin,^[^ ^12^ ^]^ colisporifungin,^[^ ^13^ ^]^ verruculin,^[^ ^14^ ^]^ ophiotine,^[^ ^15^ ^]^ and aselacin A^[^ ^16^ ^]^ (Figure S3, Supporting Information). They all share a common macrocyclic scaffold and mainly differ in the fatty acyl moiety. Some of them have been associated with various bioactivities, e.g., colisporifungin was reported to potentiate the activity of caspofungin but does not exhibit antifungal activity of its own.^[^ ^13^ ^]^ Similarly, symbiosin was reported to boost the activity of necroxime by acting as a biosurfactant while not exhibiting nematicidal activity of its own,^[^ ^12^ ^]^ whereas ophiotine was reported to exhibit moderate nematicidal activity on its own.^[^ ^15^ ^]^ The antimicrobial capacity of **1** was evaluated by assaying germination inhibition of various filamentous fungi as well as growth inhibition of the pathogenic yeast *Candida albicans*, the oomycete *Phytophthora infestans*, and various bacteria (**Table** **1**). Intriguingly, **1** almost selectively inhibited germination of *B. cinerea* at an MIC of 3 μg mL^−1^ (≈3.2 μM). Germination of *Magnaporthe oryzae* was also partially inhibited however only when assayed in H_2_O but not when assayed in media.

**Table 1:** Antimicrobial activity of 1.

Organism	MIC [μg mL^−1^]
*Magnaporthe oryzae* (CM/H_2_O)a)	–c)/>100 d)
*Botrytis cinerea* a)	3 (≈3.2 μM)
*Fusarium graminearum* a)	–
*Aspergillus oryzae* a)	–
*Candida albicans* b)	–
*Phytophthora infestans* b)	–
*Staphylococcus aureus*	–
*Pseudomonas aeruginosa*	–
*Aneurinibacillus migulanus* b)	–
*Enterobacter cloacae* subsp. *dissolvens* b)	–

a) Germination inhibition;

b) Vegetative growth inhibition;

c) No activity up to 100 μg mL^−1^;

d) Partially inhibited even at 5 μg mL^−1^, but not fully inhibited even at 100 μg mL^−1^.

When treated with **1**, even at sub‐MIC, conidia of *B. cinerea* appear to swell to ≈1.5‐fold their usual size (Figure S4, Supporting Information). This has previously been observed when conidia of *B. cinerea* were treated with caspofungin,^[^ ^17^ ^]^ an echinocandin‐type cyclic lipodepsipeptide fungicide known to interfere with cell wall biogenesis by noncompetitively inhibiting the 1,3‐β glucan synthase. It is therefore conceivable that the mode of action of **1** might be the inhibition of cell wall biogenesis in *B. cinerea*. As this effect would not be specific to conidia germination but detrimental to the fungus in general, an agar disc assay was performed (Figure S5, Supporting Information), which confirmed that **1** also inhibits the vegetative growth of *B. cinerea*. Additionally, compound **1** exhibits surfactant properties similar to symbiosin (Figure S6, Supporting Information);^[^ ^12^ ^]^ therefore, it might additionally be capable of disrupting membrane integrity and/or permeability. The exact mechanism of action of **1** and particularly the selective susceptibility of *B. cinerea* remain elusive and yet to be determined. Furthermore, the physiological function of **1** is also unclear, especially as it is mostly retained in the mycelium of the fungus.

While all the other compounds addressed are of fungal origin, symbiosin is produced by symbiotic endofungal bacteria instead. A plausible candidate biosynthetic gene cluster (BGC) was proposed for symbiosin biosynthesis based on genome mining and in silico analysis; however, validation thereof was not possible as the symbionts were unculturable.^[^ ^12^ ^]^ In contrast, the biosynthetic pathways of the related fungal cyclic lipodepsipeptides have not been investigated at all, and therefore, we set out to representatively examine the biosynthesis of **1**. To this end, the genome of the fungal strain IBWF 020‐21 was sequenced and assembled into 38.3 Mb. The assembly comprised 172 contigs, with an N50 of 0.49 Mb and a GC content of 48.4%. The length of the largest contig was 1.4 Mb. A total of 5 110 840 clean reads were obtained from Illumina sequencing, enabling the prediction of 8158 protein‐coding genes with functional annotations. AntiSMASH^[^ ^18^ ^]^ and FunBGCeX^[^ ^19^ ^]^ analyses together detected 55 secondary metabolite BGCs including 12 NRPS BGCs, 9 polyketide synthase (PKS) BGCs, 1 hybrid PKS/NRPS BGC, 12 terpene synthase (TPS) BGCs, 15 ribosomally synthesized and post‐translationally modified peptide (RiPPs) BGCs, 4 isocyanide synthase (ICS) BGCs, and 2 UbiA‐type prenyltransferase (PT) BGCs.

**Figure 2:**
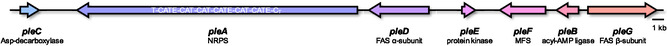
*ple* biosynthetic gene cluster.

Among the NRPS BGCs, a single‐candidate BGC likely responsible for the biosynthesis of **1** was identified. The *ple* cluster (accession number: PV294983) encodes a hexamodular NRPS PleA, an acyl‐AMP ligase PleB, an aspartate decarboxylase PleC, a fatty acid synthase (FAS) α‐subunit PleD, a putative protein kinase PleE, an ABC transporter PleF, and a FAS β‐subunit PleG (**Table** **2**, **Figure** **2**). The structure and stereochemistry of **1** are in accord with the number of modules and the arrangement of E‐domains in PleA. Moreover, there is an additional T_0_‐domain in PleA that is likely required for loading of the myristoyl moiety onto the assembly line. PleB is homologous to GloD, the acyl‐AMP ligase involved in pneumocandin biosynthesis.^[^ ^7^ ^]^ GloD catalyzes lipoinitiation by activating polyketide synthase (GloL)‐derived 10,12‐dimethyl‐myristic acid and transferring it to the T_0_‐domain of GloA. Interestingly, abolishing the production of 10,12‐dimethylmyristic acid via deletion of *gloL* enabled the production of pneumocandin derivatives with alternative fatty acyl moieties, among others MA, suggesting GloD to exhibit some substrate tolerance.^[^ ^7^ ^]^ This strengthens the hypothesis that PleB catalyzes lipoinitiation in the biosynthesis of **1**, activating and transferring MA to the T_0_‐domain of PleB. As palmitic and linoleic acid are the most abundant fatty acids in various acsomycetes,^[^ ^7^, ^20^ ^]^ the FAS subunits PleD and PleG likely collaborate to provide sufficient amounts of MA for the biosynthesis of **1**. PleC is likely to provide β‐alanine for the biosynthesis of **1**, as it is homologous to DtxS4, an l‐aspartate decarboxylase that converts l‐aspartate to β‐alanine for the biosynthesis of destruxins.^[^ ^21^ ^]^

**Figure 3:**
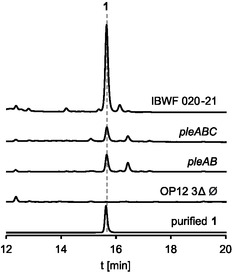
Heterologous reconstitution of pleosporacin (**1**) biosynthesis in *Aspergillus oryzae* OP12. Chromatograms (280 nm) of mycelia extracts of OP12 mutant strains, IBWF 020‐21 and purified **1**.

**Scheme 1:**
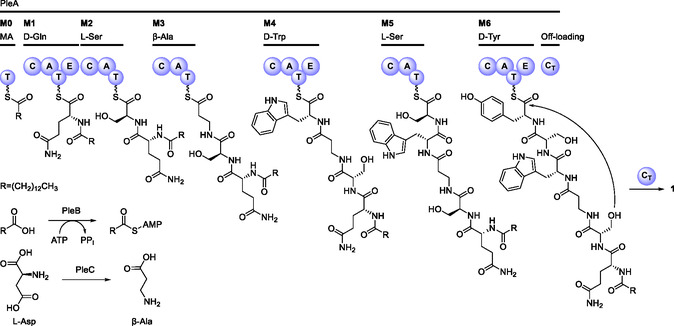
Proposed biosynthesis of pleosporacin (**1**).

Multimodular NRPS are often challenging to characterize, particularly due to their enormous size and associated difficulties during cloning. Therefore, the biosynthetic pathways of large fungal NRPs are frequently elucidated through gene deletions in native producers instead.^[^ ^6^, ^21^, ^22^ ^]^ This methodology is, however, limited to organisms that are amenable to genetic manipulation. Consequently, various techniques have been employed to facilitate cloning for subsequent heterologous characterization, ranging from in vivo assembly of plasmids^[^ ^23^ ^]^ or amplicons^[^ ^24^ ^]^ to sequential ligation cloning.^[^ ^25^ ^]^ Due to the slow growth of IBWF 020‐21 we also attempted and succeeded in sequentially assembling the ≈25 kb NRPS gene *pleA* into a plasmid for heterologous expression using multistep Gibson assembly. *pleA*, *pleB*, and *pleC* were introduced into the heterologous host *Aspergillus* *oryzae* OP12 3Δ^[^ ^26^ ^]^ for subsequent metabolite analysis (**Figure** **3**). Indeed, the coexpression of *pleABC* resulted in autonomous production of **1**, albeit at a lower yield as compared to IBWF 020‐21. This discrepancy is likely due to a difference in the abundance of the precursor molecule MA. In the original producer IBWF 020‐21, MA is likely efficiently provided for the biosynthesis of **1** in a spatiotemporal manner via the action of the FAS encoded by *pleD* and *pleG*. Additionally, multiple other compounds similar in *m/z* and UV were produced by OP12_*pleABC* and also IBWF 020‐21 (Figure S7, Supporting Information). These are likely congeners of **1** with altered fatty acyl moieties that derive from promiscuous activity of PleB. Contrary to expectation, the coexpression of solely *pleAB* also results in the autonomous production of **1**. Prototrophic organisms inherently produce β‐alanine, as it is, e.g., part of pantothenic acid (vitamin B_5_), which is essential for multiple cellular processes including synthesis of coenzyme A. OP12_*pleAB* is apparently able to utilize the intrinsic pool of β‐alanine for production of **1**. PleC likely boosts β‐alanine supply for higher level production of **1** in the native producer, which is not achieved in OP12_*pleAB* and OP12_*pleABC*, presumably due to insufficient MA supply. Based on these findings the biosynthesis of **1** is proposed to proceed as depicted in **Scheme** **1**. The FAS α‐ and β‐subunits PleD and PleG collaborate to synthesize MA, which is subsequently activated by the fatty acyl‐AMP ligase PleB and transferred to the T_0_ domain of the NRPS PleA. The peptide chain is then elongated with d‐Gln_1_, l‐Ser_2_, β‐Ala_3_ – which is derived from PleC‐catalyzed decarboxylation of l‐Asp – d‐Trp_4_, l‐Ser_5_, and d‐Tyr_6_. Finally, macrolactonization between the β‐hydroxyl moiety of l‐Ser_2_ and the α‐carbonyl of d‐Tyr_6_ is catalyzed by the terminal C_T_‐domain, resulting in the release of **1** from the assembly line.

**Table 2:** Proteins encoded in the *ple* cluster.

Gene	Length [aa]	BLASTp^a^^)^	Identity [%]/*E*‐value	Proposed function
*pleC*	519	E9FCP7.2—l‐aspartate decarboxylase dtxS4 [*Metarhizium robertsii*]	44.93/3 E^−151^	l‐Asp decarboxylase
*pleA*	8396	E9FCP4.2—Nonribosomal peptide synthetase dtxS1 [*Metarhizium robertsii*]	42.81/0.0	NRPS
*pleD*	1665	P15368.1—Fatty acid synthase subunit alpha [*Penicillium griseofulvum*]	46.57/0.0	FAS α‐subunit
*pleE*	373	P36615.1—Serine/threonine‐protein kinase csk1 [*Schizosaccharomyces pombe*]	25.15/2 E^−04^	Protein kinase
*pleF*	1281	Q4WTT9.1—ABC‐type transporter MDR1 [*Aureobasidium melanogenum*]	49.84/0.0	ABC transporter
*pleB*	481	S3DB78.1—Acyl‐CoA ligase gloD [*Glarea lozoyensis*]	38.29/7e^−133^	Fatty acyl‐AMP ligase
*pleG*	2047	P34229.2—Fatty acid synthase subunit beta [*Yarrowia lipolytica*]	42.80/0.0	FAS β‐subunit

a) Nearest fungal hit using Uniprot database.

The production of similar cyclic lipodepsipeptides in fungi and bacteria might be indicative of a horizontal gene transfer event. However, the GC content of the *ple* BGC (48.8%) and *pleA* (50.8%) are well in accord with the overall genomic GC content (48.4%). Moreover, PleA and the symbiosin NRPS^[^ ^12^ ^]^ differ in domain architecture (Figure S8, Supporting Information) and employ different strategies for lipoinitiation and terminal cyclization. Therefore, it can be presumed that the *ple* BGC is truly of fungal origin and was unlikely acquired via horizontal gene transfer.

## 3 Conclusion

In search for antifungal compounds, we isolated the cyclic lipodepsipeptide pleosporacin (**1**), a new selective inhibitor of *B. cinerea*, from mycelia of the fungal strain *Plepsporales* sp. IBWF 020‐21. Although the mechanism remains elusive yet, compound **1** is hypothesized to interfere with the cell wall biogenesis of *B. cinerea*. The discovery and development of more selective antimicrobial agents are crucial for a greener agriculture, as they exhibit fewer nontarget side effects, easing the environmental impact while not compromising crop protection. By investigating the biosynthesis of **1**, it is demonstrated that it is possible and feasible to clone large genes for heterologous characterization, in cases where homologous investigations are not possible. To our knowledge, this is the first characterized biosynthetic pathway of a cyclic lipodepsipeptide related to aselacin A.

## Conflict of Interest

The authors declare no conflict of interest.

## Supporting information

Supplementary Material

## Data Availability

The data that support the findings of this study are available in the supplementary material of this article.
