# Supplementary material for: Biosynthesis of the Fungal Cyclic Lipodepsipeptide Pleosporacin, a New Selective Inhibitor of the Phytopathogen Botrytis cinerea
Source: Chembiochem. 2025 May 23;26(12):e202500315. doi: 10.1002/cbic.202500315 (PMC12177691; doi:10.1002/cbic.202500315)
Supplement: Supplementary file 1 — Supplementary Material [file CBIC-26-e202500315-s001.pdf]

Supporting Information

Biosynthesis of the fungal cyclic lipodepsipeptide pleosporacin, a new selective inhibitor of the phytopathogen *Botrytis cinerea*

Carsten Wieder*[a,b], Rainer Wiechert[c], Alexander Yemelin[b], Louis Pergaud Sandjo[d], Eckhard Thines[a,b], Till Opatz[c], Anja Schüffler*[b]

[a] Institute of Molecular Physiology, Johannes Gutenberg-University, Hanns-Dieter-Hüsch Weg 17, D-55128 Mainz, Germany

[b] Institut für Biotechnologie und Wirkstoff-Forschung gGmbH, Mainz, Hanns-Dieter-Hüsch Weg 17, D-55128 Mainz, Germany

[c] Department of Chemistry, Johannes Gutenberg-University, Duesbergweg 10–14, D-55128 Mainz, Germany

[d] Department of Chemistry, Universidade Federal de Santa Catarina, Florianópolis 88040-900, Santa Catarina, Brazil

*Correspondence: cawieder@uni-mainz.de, schueffler@ibwf.de

Table of Contents

I. Experimental procedures 2

II. Supplementary Figures 7

III. Analytical data 11

IV. ^{1}H- and ^{13}C{^{1}H}-NMR Spectra 14

V. References 11

I. Experimental Procedures

Fungal strains

Fungal strain IBWF 020-21 was isolated from a freshwater sediment sample taken from the Elbe river (near shoreline, sediment plus freshwater) in Hamburg, Germany, in July 2021 by serial dilution plating on Littmann (1.5 % oxgall, 1 % peptone, 1 % glucose, 0.01 g crystal violet, pH 7.0) agar plates supplemented with 100 µg/mL streptomycin. The strain is deposited at the Institut für Biotechnologie und Wirkstoff-Forschung gGmbH (IBWF), Mainz, Germany and was routinely maintained on YMG (0.4 % yeast extract, 1 % malt extract, 1 % glucose, pH 5.5) agar plates at room temperature. *Aspergillus oryzae* OP12 3Δ was routinely cultivated on GG10 (50 mM glucose, 10 mM glutamine, 0.52 g/L KCl, 0.52 g/L $MgSO_4 \cdot 7\ H_2O$, 1.52 g/L KH_2PO_4; 1 mL/L Hutner's trace elements; pH 6.5) agar plates supplemented with10 mM uridine, 0.0001 % *p*-amino benzoic acid (PABA) and 0.1 % arginine at 30 °C for sporulation and maintained as spore suspension in PBS at 4 °C. All mutant strains used in this study are listed in Table S1.

Table S. 1: Mutant strains used in this study

Mutant	Parental Strain	Genotype	Source
OP12 3Δ	see source	*PamyB:terR_ptrA*; *pyrG*⁻, *ΔpabA*, *ΔargB*	[1]
OP12 empty plasmid control	OP12 3Δ	*PamyB:terR_ptrA*	[1]
OP12_*pleAB*	OP12 3Δ	*PamyB:terR_ptrA*, *PterA:pleA_URA*, *PterA:pleB_argB*, *ΔpabA*	This study
OP12_*pleABC*	OP12 3Δ	*PamyB:terR_ptrA*, *PterA:pleA_URA*, *PterA:pleB_argB*, *PterA:pleC_pabA*	This study

ITS and whole genome sequencing

Genomic DNA of IBWF 020-21 was prepared from lyophilized mycelium using the GeneJET Plant Genomic DNA Purification Kit (ThermoFisher) according to the manufacturer's instructions. The ITS barcode region was amplified using the ITS1F and ITS4 primers with the Phire Green Hot Start II PCR Mastermix (Thermo Fisher), purified using the Monarch PCR & DNA Cleanup Kit (NEB) and analyzed by Sanger sequencing. The consensus of forward and reverse sequencing was BLASTed against the NCBI ITS database.

Table S. 2: ITS sequence of IBWF 020-21

Fungal strain	ITS sequence	selected BLAST hits	Identity/E-value
IBWF 020-21	AGTAAAAGTCGTAACAAGGTTTCCGTAGGTGAACCTGCGGAAG<br>GATCATTATCAATTTACAGCGGACTTCGGTCCTGTCTGCACCC<br>TTGTCTTTTGCGTACTGTATGTTTCCTTGGTAGGCTTGCCTAC<br>CAATAGGACATCATAAACTCTTTTGTAATTGCAATCAGCGTCA<br>GAAAACTATAATAGTTACAACTTTCAACAACGGATCTCTTGGT<br>TCTGGCATCGATGAAGAACGCAGCGAAATGCGAAAAGTAGTGT<br>GAATTGCAGAATTCAGTGAATCATCGAATCTTTGAACGCACAT<br>TGCGCCCCTTGGTATTCCATGGGGCATGCCTGTTCGAGCGTCA<br>TTTGTACCCTCAAGCTCTGCTTGGTGTTGGGTGTTTGTCCCGC<br>TTATACGCGTGGACTCGCCTTAAAGCAATTGGCAGCCGGCATA<br>CTAGCCTGGGAGCGCAGCACATTTTGCGTTTCTTGCTTTGAAT<br>GTCGACGTCCATCAAGTCAAACATTTTGCTCTTGACCTCGGAT<br>CAGGTAGGGATACCCGCTGAACTTAAGCATATC	GU327433.1<br>uncultured fungus	99.82%/0.0
		MF795792.1<br>*Pyrenochaeta nobilis*<br>CBS 407.76	94.75%/0.0
		NR_156358.1<br>*Neocucurbitaria juglandicola*<br>CBS142390	92.25%/0.0

Whole genome sequencing was performed on Illumina HiSeq 2500 platform resulting in 150 bp paired-end reads. The integrity and quality of raw sequence data were initially assessed using FastQC (v0.12.1)[2]. Reads were subsequently filtered to remove low-quality sequences. De novo assembly was performed using SPAdes (v3.15.2) with k-mer lengths of 77, 99, and 121, along with the --careful and --only-assembler parameters to minimize assembly errors[3]. The assembly command used was as follows:

```
/opt/anaconda3/envs/de_novo_assembly/bin/spades.py -1 IBWF/1.fastq.gz -2 IBWF/2.fastq.gz
-o IBWF/spades_kmers_set_careful_assembly -t 4 -k 77,99,121 --careful –only-assembler
```

Quality assessment was performed by the QUAST (v5.0.2) tool[4].The assembly with the highest contiguity, as indicated by the largest N50 and contig lengths, was selected for downstream analyses. Gene prediction was carried out using Augustus with a pretrained fungal models. Functional annotation of predicted genes included BLAST comparisons against Swiss-Prot and InterPro databases. Genome visualization was performed using IGV (v2.14.0)[5]. Secondary metabolite biosynthetic gene clusters (BGCs) detection were carried out using antiSMASH (v7.1.0)[6] and FunBGCeX[7]. Predicted clusters were manually curated and validated by BLASTp comparisons against the NCBI non-redundant protein database to confirm functional annotation and assess cluster architecture.

Construction of plasmids and mutants

pleB and *pleC* were amplified from genomic DNA of IBWF 020-21 using Q5 Hot Start High-Fidelity 2X Master Mix (NEB) and assembled into *Nco*I-restricted SM-Xpress_argB(mut) and SM-Xpress_pabA, respectively, using the HiFi DNA assembly master mix (NEB). The ~25 kb CDS of *pleA* was sequentially assembled into SM-Xpress_Ura in three successive HiFi DNA assembly reactions. Initially, four (of seven) fragments were amplified and assembled into *Nco*I-restricted SM-Xpress_Ura, inserting a *Psp*OMI restriction site after the final fragment for reopening the intermediate plasmid after assembly. We sequenced the resulting plasmid and noticed only partial integration of the fourth fragment, however the *Psp*OMI restriction site was intact. Therefore, we redesigned a primer for amplification of the fourth fragment accounting for the new overlap and inserted new fragment four and fragment five into the *Psp*OMI-restricted intermediate plasmid, again inserting a new *Psp*OMI after the fifth fragment. Finally, fragments six and seven were assembled into the *Psp*OMI-restricted second intermediate plasmid and the integrity of the final plasmid validated by whole plasmid sequencing. All oligonucleotides used in this study are listed in Table S3. For construction of OP12_*pleAB* and OP12_*pleABC*, protoplast transformation of OP12 3Δ was carried out as previously described[1]. Integration of *pleB* and *pleC* was confirmed by diagnostic PCR using the 2× Phire Green Hot Start II PCR Master Mix (Thermo) (Figure S. 9).

Table S. 3: Oligonucleotides used in this study

Oligo	Sequence	Purpose
oCW282	catttaacaaacttctcatcacagcaccatgaatgaaataggtgaaagag	Amplification of *pleA* fragments for sequential cloning of SMX_*pleA*_Ura
oCW283	gtgcatcgcgtttatcgtca	
oCW347	gcggtcttcctacaggttga	
oCW522	ctcagtcggatcctgacctt	
oCW523	attgaacttccgttttcagg	
oCW350	tggctgggatatgacttgct	
oCW288	tattcacgacctgatcgctg	
oCW296	ggttcagattgaaatcactgctgcgggcccacttgctagcctagtcgaag	
oCW530	agatgacgtacaacgagcta	
oCW351	ttacaatgagctggatgaaa	
oCW531	ggttcagattgaaatcactgctgcgggcccctttaacatacctcggttgg	
oCW525	gtttccaggaaggtcaagat	
oCW526	tagtgaggatctcatcaata	
oCW294	tcctacgctttcgatgcttg	
oCW295	ctatacggttcagattgaaatcactgctgctcaagtaaatgctggcaag	
oCW549	catttaacaaacttctcatcacagcaccatggtcttcactactcccgc	Amplification of *pleB* fragments for cloning of SMX_*pleB*_argB(mut)
oCW313	ccactcacgatattgtccac	
oCW314	gtggacaatatcgtgagtgg	
oCW298	ctatacggttcagattgaaatcactgctgcctacggcatctcattcgac	
oCW299	Catttaacaaacttctcatcacagcaccatggttgtagaaaagtttccta	Amplification of pleC for cloning of SMX_*pleC*_paba
oCW300	Ctatacggttcagattgaaatcactgctgcctacattatgaaggactgg	
oCW75	gcgcaaagacacatgatg	Used for diagnostic PCRs, anneals in the P*terA* promoter
ITS1F	cttggtcatttagaggaagtaa	Amplification of the ITS barcode region
ITS4	tcctccgcttattgatatgc	

Fermentation, extraction, HPLC-MS analysis, extract fractioning and isolation of pleosporacin

In an initial screening, fungal strain IBWF 020-21 was cultivated in 500 mL of YMG media (0.4 % yeast extract, 1 % malt extract, 1 % glucose) for 20 days shaking at 120 rpm at rt. OP12 mutant strains were cultivated in 50 mL 2 % starch media (2 % soluble starch, 10 mM glutamine, 0.52 g/L KCl, 0.52 g/L $MgSO_4 \cdot 7\,H_2O$, 1.52 g/L KH_2PO_4; 1 mL/L Hutner's trace elements; pH 6.5) for 3 days shaking at 150 rpm at rt. Mycelia was harvested by vacuum filtration and extracted with MeOH:actetone (1:1) shaking for 30-60 minutes at rt. The suspension was filtered and dried under reduced pressure. Crude extracts were redissolved in MeOH and analyzed by LC-MS using a LiChrospher 100 RP-18 column (125 mm × 2 mm, 4 µm, Merck KGaA) connected to Agilent DAD 1260 and Quadrupole LC/MS 6130 modules for detection, running a gradient of 1 to 100 % of ACN in H_2O + 0.1 % formic acid in 20 minutes at a flow rate of 0.4 mL/min.

To identify the active ingredient in the mycelia extract of IBWF 020-21, the extract was fractioned using HPLC. The resulting fractions were then re-assessed for germination inhibitory activity against *B. cinerea* and only fractions containing compound **1** exhibited activity.

For isolation of compound **1**, IBWF 020-21 was cultivated in a 20 L bioreactor (YMG media) stirring at 120 rpm at room temperature for 9 days. The mycelium was separated from the culture broth and lyophilized. The mycelium (132 g) was subsequently extracted with 2 L MeOH:actetone (1:1) overnight three times. The dried crude extract (25.6 g) was prefractionated using silica gel chromatography (CH→EE→MeOH; target fraction: 3.1 g) and SPE (Bond Elut C18 Cartridge, Agilent, H_2O→ACN). Half of the SPE fraction containing most **1** (65% ACN fraction, 0.46 g total) was applied to preparative HPLC on a Sunfire C18 column (100 Å, 5 µm, 19 mm × 250 mm, Waters GmbH) running at 17 mL/min (isocratic elution, 62 % ACN, 38 % H_2O + 0.1 % formic acid), yielding 54.4 mg of pure **1** as an off-white amorphous solid.

Analytical chemistry for structure elucidation

Thin layer chromatography

Analytical thin-layer chromatography (TLC), 0.25 mm silica plates (60 F254) from Merck were used, and the detection was reached by fluorescence quenching under UV light (λ = 254 nm) or by staining with potassium permanganate reagent (solution of $KMnO_4$ (3 g), K_2CO_3 (20 g), 5 % NaOH (5 mL), and H_2O (300 mL)) followed by heating to 400 °C.

NMR spectra

Measured NMR spectra were, unless otherwise mentioned, recorded at 296 K on a 600 MHz Bruker Avance-III 600 spectrometer with a 5 mm TCI cryoprobe. After prior referencing to the residual solvent signal (Acetone-d_6: 2.05 & 29.84 ppm for ^{1}H NMR and ^{13}C NMR, respectively), all chemical shifts (δ) are reported relative to residual solvent[8]. Coupling constants were reported in Hz and the signal multiplicities were abbreviated as follows: s (singlet), d (doublet), t (triplet), q (quartet), qd (quartet of doublet), m (multiplet), br (broad).

Infrared spectra

Infrared spectroscopy was performed on a Bruker Tensor 27 FTIR spectrometer including a diamond ATR unit and are reported in terms of absorption frequency $\bar{\nu}$ [cm^{-1}].

Mass spectra

HRMS and MS/MS were conducted on an Agilent G6545A Q-ToF with ESI, APCI or APPI source coupled with an Agilent 1260 Infinity II HPLC system. If not described otherwise, spectra were recorded using positive ionization mode.

Optical rotations

Optical rotation measurements were accomplished with a Perkin-Elmer 241MC polarimeter at λ = 589nm. A solvent-filled cuvette was used for instrument calibration[9].

Marfey analysis for determining the stereochemistry of pleosporacin

Marfey analysis was adapted from Büttner *et al.*[10]. Briefly, 1 mg of **1** was hydrolyzed in 2 mL of 6 M HCl at 110 °C overnight or alternatively for 1 h only to prevent degradation of tryptophan[11]. The hydrolysate was dried under a N_2 stream and subsequently redissolved in 500 µL 1 M $NaHCO_3$. 50 µL of a 1 % solution of L-FDAA in acetone was added to 100 µL of the hydrolysate and the reaction heated to 50 °C for 1 h. The reaction was quenched by the addition of 50 µL of 2 M HCl and subsequently diluted with 200 µL of ACN/H_2O (1:1). 200 µg of each amino acid (D- and L-, Gln, Ser, Trp, Tyr) were derivatized in the same way. 5 µL of the references or 20 µl of the **1** hydrolysate were analyzed using LC-MS, running a gradient of H_2O + 0.1 % formic acid and ACN as follows: 1–25 % ACN in 30 min, isocratic 25 % ACN for 10 min, 25–70 % ACN in 20 min, 70–100 % ACN in 1 min, isocratic 100 % ACN for 4 min. Stereochemistry was assigned by comparison of the hydrolysate and amino acid reference chromatograms.

Bioactivity Assays

Germination inhibition of ascomycete fungi

Conidia of *Magnaporthe oryzae* 70-15, *Botrytis cinerea* DSM 0877, *Fusarium graminearum* DSM 21727 and *Aspergillus oryzae* RIB40 were harvested from agar plates and diluted in 2 % malt extract media to a final concentration of $1x10^5$ conidia/mL. 200 µL of the solution were added to wells of a 96-well plate containing different concentrations of the purified compounds. The plates were then incubated overnight at room temperature. Conidia germination was evaluated using a microscope. Ciclopirox (100 µg/mL) served as positive control.

Vegetative growth inhibition of Botrytis cinerea

For assessing whether **1** was fungicidal or merely inhibited germination of *B. cinerea*, antibiotic assay discs were placed on a YMG agar plate and 20 µg, 10 µg, 5 µg, 1 µg **1** or MeOH were added to them. Then, *B. cinerea* was spotted in the middle of the plate and allowed to grow for 7 days at rt. The growth inhibitory activity was visually assessed.

Growth inhibition of dimorphic yeast Candida albicans

Candida albicans ATCC90028 was grown on Sabouraud (Difco) plates. Fresh colonies were suspended in H_2O, diluted 1:20 in Sabouraud media, 200 µL distributed in 96-well test plates and cultivated shaking at room temperature for 18–24h; growth inhibition was assessed macroscopically. Ciclopirox (100 µg/mL) served as positive control.

Growth inhibition of oomycete Phytophthora infestans

2 mL of a 2-week-old liquid PDA (Difco) culture of *Phytophthora infestans* CBS 430.90 were shredded using a FastPrep twice for 20 s, diluted with 5 mL of H_2O and filtered through miracloth. The filtrate was diluted 1:20 with KGA media and 200 µL distributed in 96-well test plates. Plates were incubated gently shaking at room temperature for 1 week; growth inhibition was assessed macroscopically. Ciclopirox (100 µg/mL) served as positive control.

Growth inhibition of bacteria

Nutrient broth (Difco) precultures of *Staphylococcus aureus* ATCC11632 (37 °C), *Pseudomonas aeruginosa* ATCC15442 (37 °C), *Aneurinibacillus migulanus* ATCC9999 (37 °C) and *Enterobacter cloacae* subsp. *dissolvens* LMG2683 (27 °C) were grown shaking overnight. Precultures were diluted 1:100 in fresh nutrient broth and 200 µL were distributed in 96-well test plates. Plates were cultivated shaking at 37 °C or 27 °C for 18–24h and growth inhibition was assessed macroscopically. Tetracycline (100 µg/mL) and Streptomycin (100 µg/mL) served as positive controls.

II. Supplementary Figures

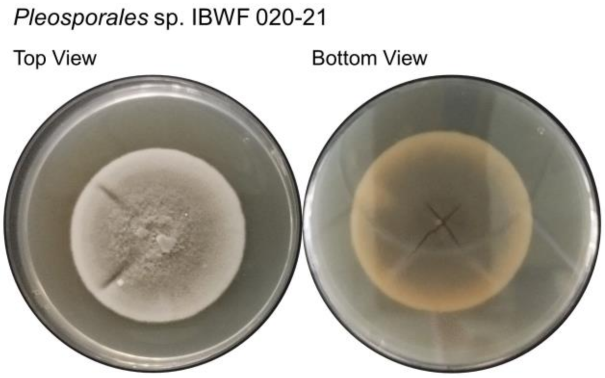

Figure S.: 1Morphology of *Pleosporales* sp. IBWF 020-21 grown on YMG media for 22 days at rt.

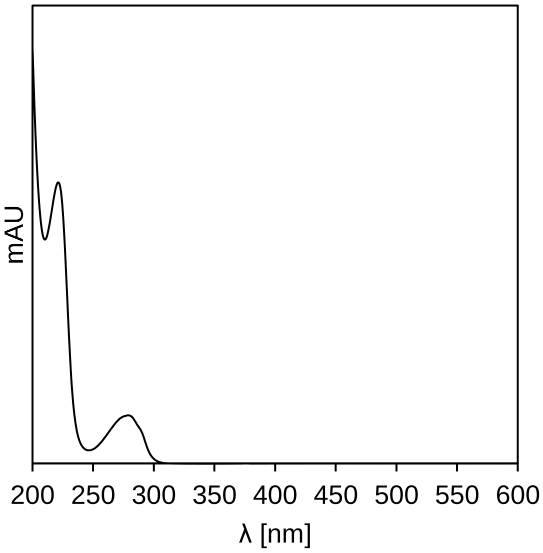

Figure S. 2: UV/Vis spectrum of pleosporacin (**1**).

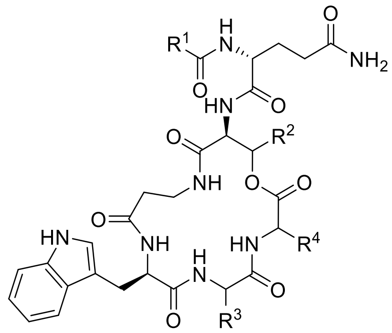

R^1=myristic acid	R^2=L-Ser	R^3=L-Ser	R^4=D-Tyr	Pleosporacin (**1**)
R^1=(*R*)-3-hydroxymyristic acid	R^2=L-Thr	R^3=L-Ser	R^4=D-Tyr	Symbiosin
R^1=dodecanoic acid	R^2=L-Thr	R^3=L-Ser	R^4=D-Phe	Colisporifungin
R^1=dodecanoic acid	R^2=L-Thr	R^3=L-Ser	R^4=D-Tyr	Verruculin
R^1=decanoic acid	R^2=L-Thr	R^3=L-Ser	R^4=D-Phe	Ophiotine
R^1=9-hydroxyoctadeca-10,12-dienoic acid	R^2=L-Thr	R^3=D-Ser	R^4=Gly	Aselacin A

Figure S. 3: Structures of pleosporacin (**1**) and related cyclic lipodepsipeptides[10,12].

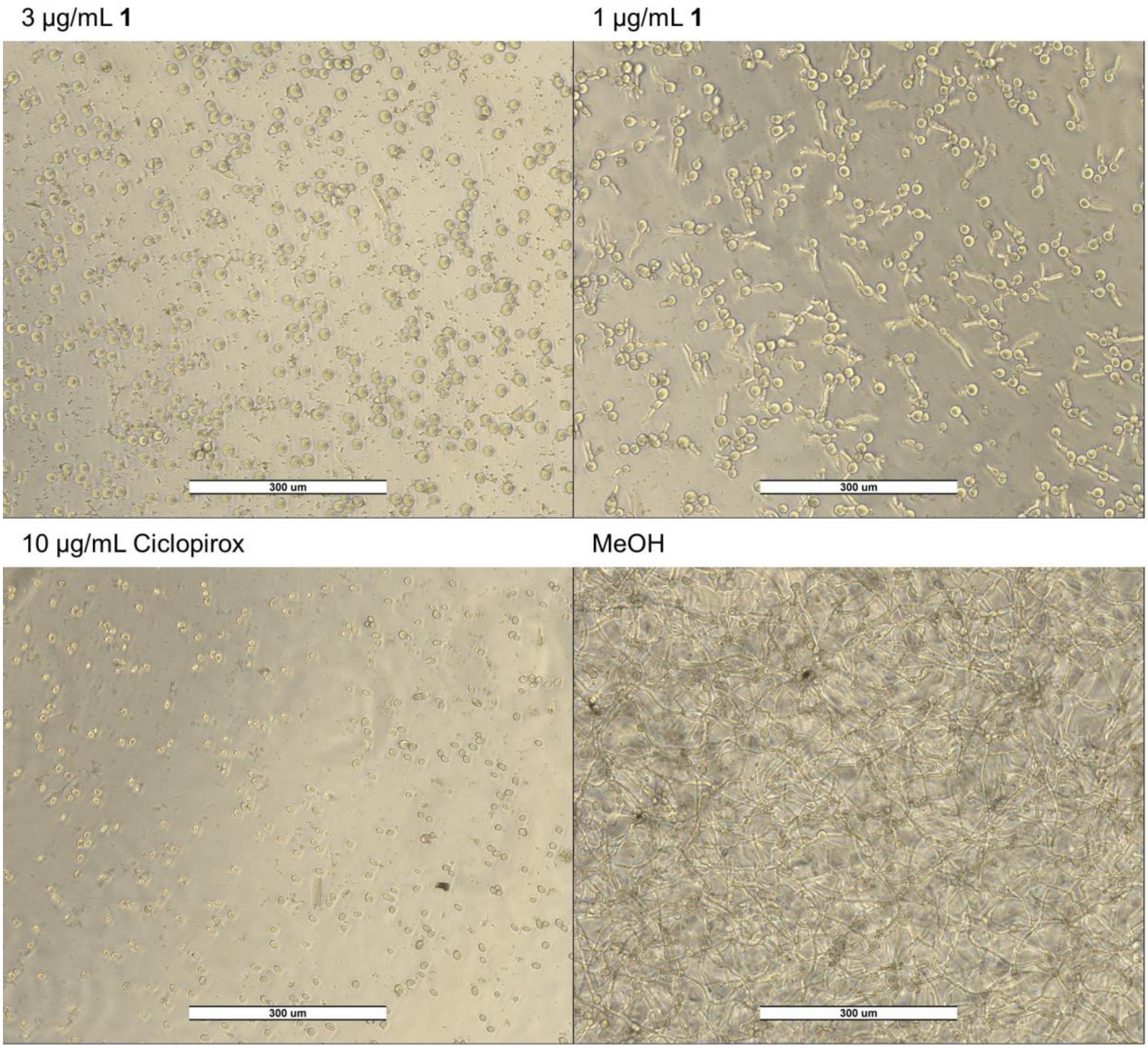

Figure S. 4: Germination inhibitory activity of **1** against *Botrytis cinerea*. Ciclopirox and MeOH served as positive and negative controls for inhibition. Sub-MIC germination is only partially inhibited, i.e., some short germ tubes are being formed. Conidia treated with **1** appear to be bloated (roughly 1.5-fold larger in size). Some media impurities are seen in the background.

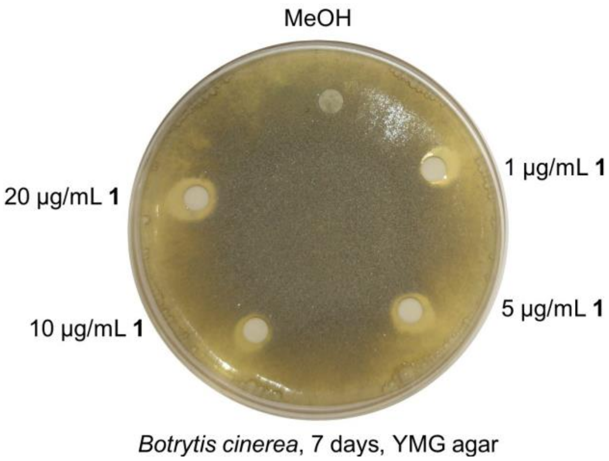

Figure S. 5: Vegetative growth inhibitory activity of **1** against *Botrytis cinerea*. Inhibition zone Ø: 8–11 mm (measured from center of the discs).

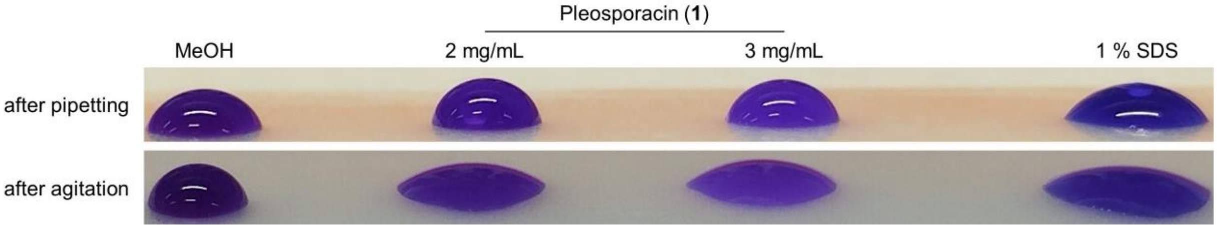

Figure S. 6: Drop Collapse Assay. Carried out as previously described by Büttner *et al.* [10]. After pipetting the drops containing pleosporacin (**1**) do not immediately collapse. However, upon agitation, the drops collapse similar to the 1 % SDS control. The surface tension of the MeOH control is not disturbed even after agitation.

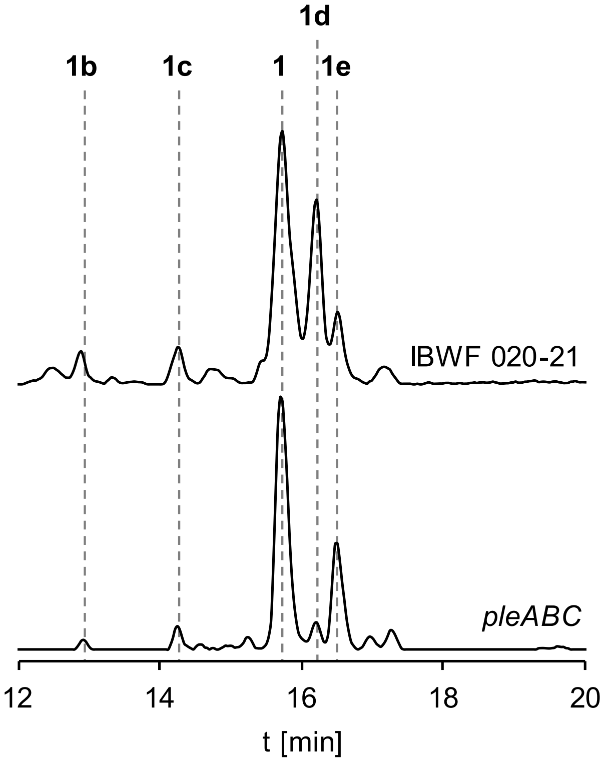

Figure S. 7: Extracted ion chromatograms (800–1000 *m/z*, recorded in negative mode) of IBWF 020-21 and OP12_*pleABC* mycelia extracts. Some of the putative **1** congeners produced are highlighted as **1b** (875 *m/z*), **1c** (903 *m/z*), **1d** (915 *m/z*) and **1e** (945 *m/z*). Presumably, these congeners harbor different length fatty acyl chains, namely (in order) decanoic acid, dodecanoic acid, tridecanoic acid and pentadecanoic acid.

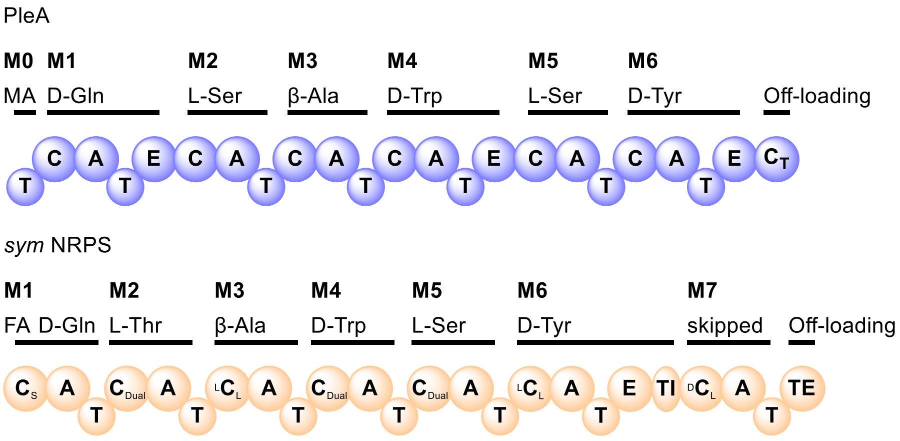

Figure S. 8: Comparison of domain architecture between PleA and *sym* NRPS (Accession No.: UUM22377.1). A, adenylation domain; C, condensation domain (S, starter; Dual, condensation/epimerization), E, epimerization domain; FA, fatty acid (3-hydroxy-myristic acid); MA, myristic acid; T, thiolation domain; TE, thioesterase domain; TI, TIGR01720 domain.

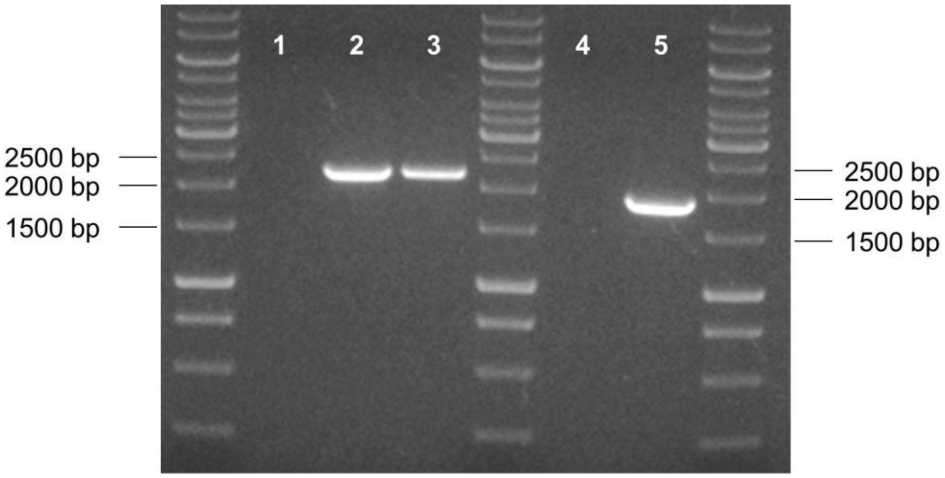

Figure S. 9: Diagnostic PCR confirming integration of *pleB* (left, Primers oCW75 + oCW298, expected amplicon: 1860 bp) and *pleC* (right, Primers oCW75 + oCW300, expected amplicon: 2163 bp). 1 and 4: OP12_empty plasmids; 2: OP12_*pleAB*; 3 and 5: OP12_*pleABC*

III. Analytical Data

Table S. 4: NMR assignment ^{1}H (600 MHz, Acetone-d$_6$) and ^{13}C NMR (151 MHz, Acetone-d$_6$)

Unit	Position	δ C, type	δ 1H, mult (Hz)	Unit	Position	δ C, type	δ 1H, mult (Hz)
D-Gln	1	171.2, q	-	TDA	1	173.4, q	-
	2	53.0, CH	4.53 (q, 7.0, 1H)		2	34.2, CH_2	2.28 –, 2.23 (t, 7.5, 2H)
	3	25.1, CH_2	2.00 – 1.95 (m, 2H)		3	24.5, CH_2	1.58 (tp, 13.8, 7.0, 2H)
	4	30.2, CH_2	2.44 – 2.29 (m, 2H)		4-11	28.6, 28.5, 28.5, 28.5, 28.5, 28.3[d]	1.26 – 1.17 (m, 16H)
	5	172.9, q	-		12	30.8	1.26 – 1.17
	NH		7.96 (d, 5.3, 1H)		13	21.5, CH_2	1.31 – 1.26 (m, 2H)
	NH2		7.02 (br, 1H)[a],6.35 (s, 1H)		14	12.5, CH_3	0.87 (t, 7.0, 3H)
L-Ser-1	1	167.8, q	-	L-Ser-2	1	169.5, q	-
	2	50.3, CH	4.75 (dt, 10.0, 2.5, 1H)		2	56.9, CH	4.19 (q, 5.5, 1H)
	3	64.2, CH_2	4.86 (dd, 10.8, 2.5, 1H), 3.85 (dd, 10.8, 2.5, 1H)		3	60.3, CH_2	3.61 (d, 11.9, 1H), 3.59 – 3.51 (m, 1H)[b]
	NH		8.65 (d, 10.0, 1H)		NH		8.10 (d, 5.6, 1H)
					Oh		3.87 (br, 1H)
β-Ala	1	171.0, q	-	D-Trp	1	173.2, q	-
	2	33.5, CH_2	2.68 (ddd, 14.5, 10.9, 4.2, 1H), 2.26 – 2.23 (m, 1H)		2	53.3, CH	4.69 (q, 7.1, 1H)
	3	36.1, CH_2	3.59 – 3.51 (m, 1H)[b], 3.11 – 3.04 (m, 1H)		3	26.8, CH_2	3.20 – 3.09 (m, 2H)[c]
	NH		7.46 (br d, 6.3, 1H)		NH	-	7.82 (s, 1H)
					NH^{Ind}	-	10.15 – 10.12 (m, 1H)
D-Tyr	1	169.5, q			2^{Ind}	122.9, CH	7.22 (d, 2.2, 1H)
	2	54.2, CH	3.76 (s, 1H)		3^{Ind}	108.5, q	-
	3	32.7, CH_2	3.20 – 3.09 (m, 2H)[c]		3a^{Ind}	126.6, q	-
	NH		7.91 (d, 7.4, 1H)		4^{Ind}	117.4, CH	7.55 (d, 7.9, 1H)
	1’	128.2, q			5^{Ind}	117.8, CH	7.03 (d, 6.1, 1H)[a]
	2’, 6’	114.0, 2C, CH	7.01 (d, 6.6, 2H)[a]		6^{Ind}	120.3, CH	7.09 (ddd, 8.1, 7.0, 1.1, 1H)
	3’, 5’	129.5, 2C, CH	6.71 – 6.66 (m, 2H)		7 ^{Ind}	110.3, CH	7.37 (dt, 8.1, 0.9, 1H)
	4’	154.9, q			7a^{Ind}	135.6, q	-
	OH	-	8.26 (s, 1H)				

[a] overlapping signals, [b] overlapping signals, [c] overlapping signals, [d] partially covered by solvent signals.

$[\alpha]_D^{21}$ +47.8 (MeOH) (c=0.23)

IR (ATR): $\tilde{\nu}$ [cm^{-1}] 3292, 2926, 2854, 1741, 1659, 1651, 1644, 1633, 1539, 1517, 1456, 1342, 1232, 1173.

LRMS (ESI) m/z (%): 933.5 (33) [M+H]$^+$.

HRMS (ESI) *m/z*: [M+Na]$^+$ Calcd for [$C_{48}H_{67}N_8NaO_{11}$]$^+$ 955.4900; Found. 955.4889.

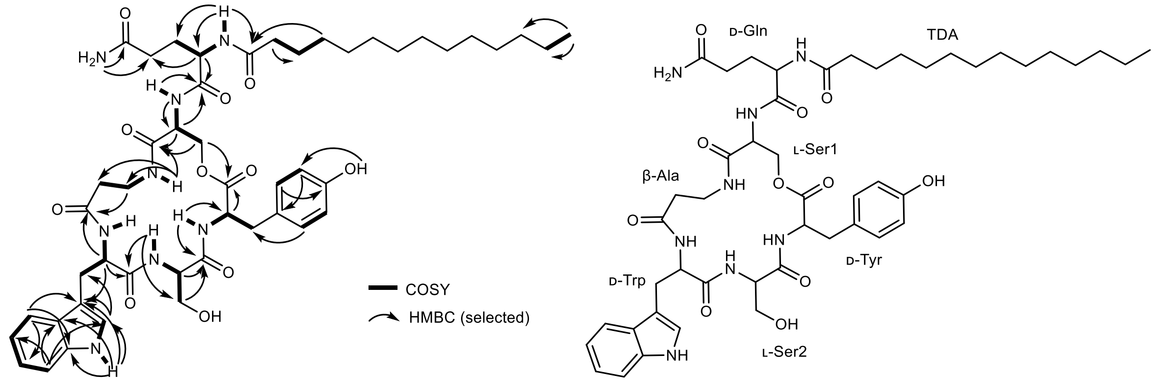

Figure S. 10: Key COSY and HMBC correlations of the NMR structure analysis.

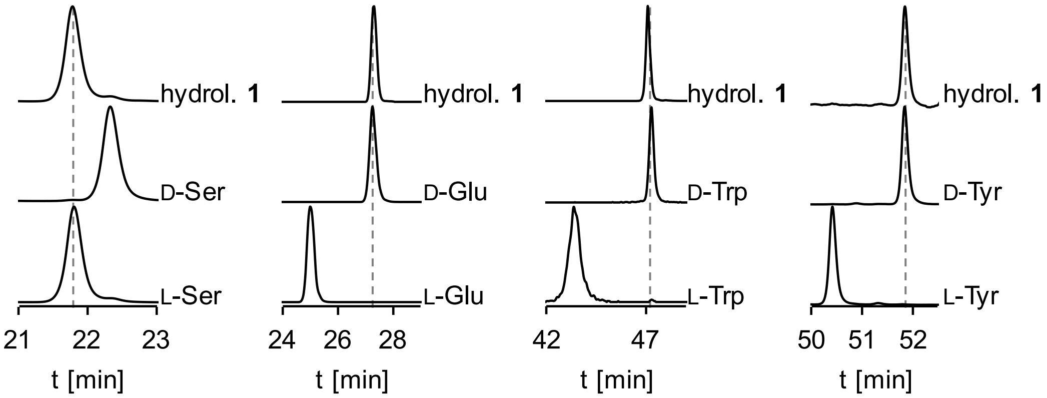

Figure S. 11: Marfey analysis for determining the stereochemistry of **1**. Gln was detected as Glu (conversion during hydrolysis). 300 nm chromatograms are shown for Ser and Tyr. EICs are shown for Glu and Trp (398 m/z and 455 m/z, respectively) due to unrelated overlapping UV/Vis peaks.

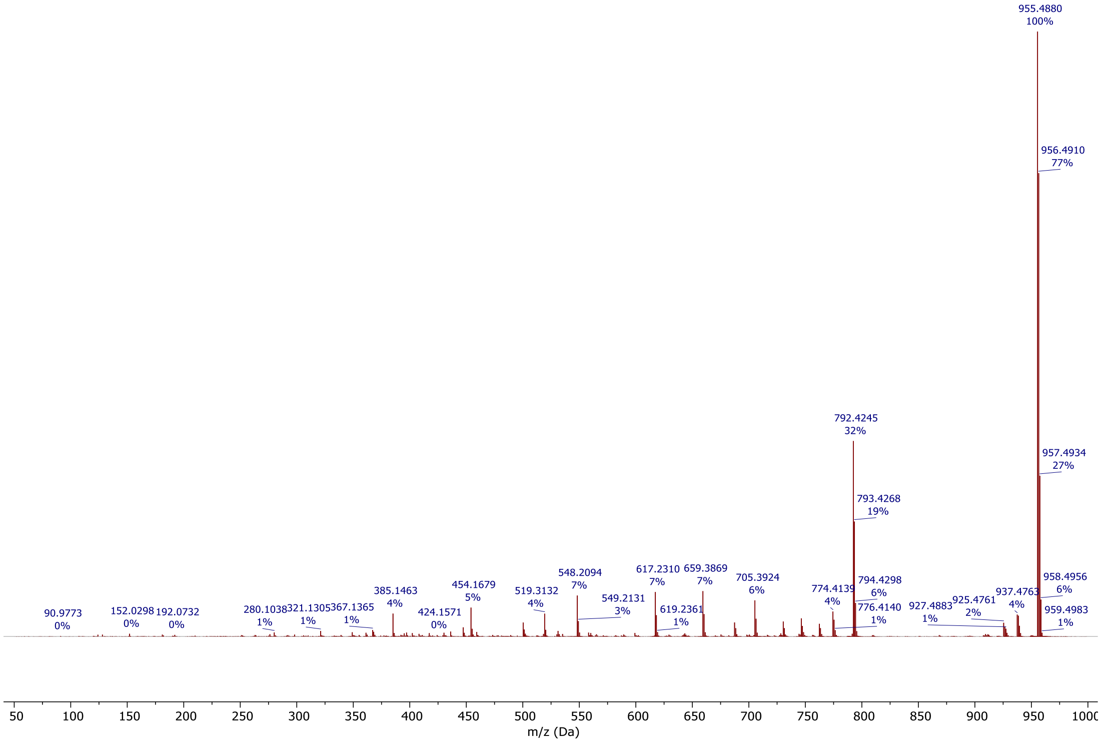

Figure S. 12: MS^2 spectrum of pleosporacin (**1**) recorded with 50 V collision energy.

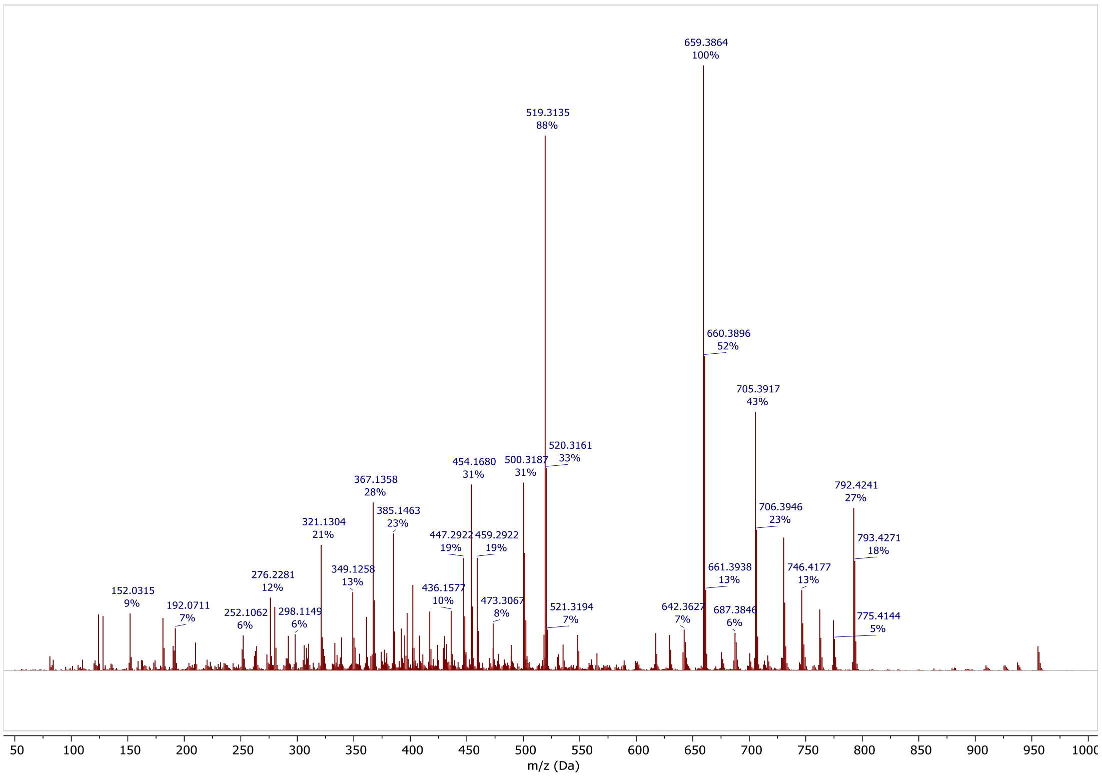

Figure S. 13: MS^2 spectrum of pleosporacin (**1**) recorded with 75 V collision energy.

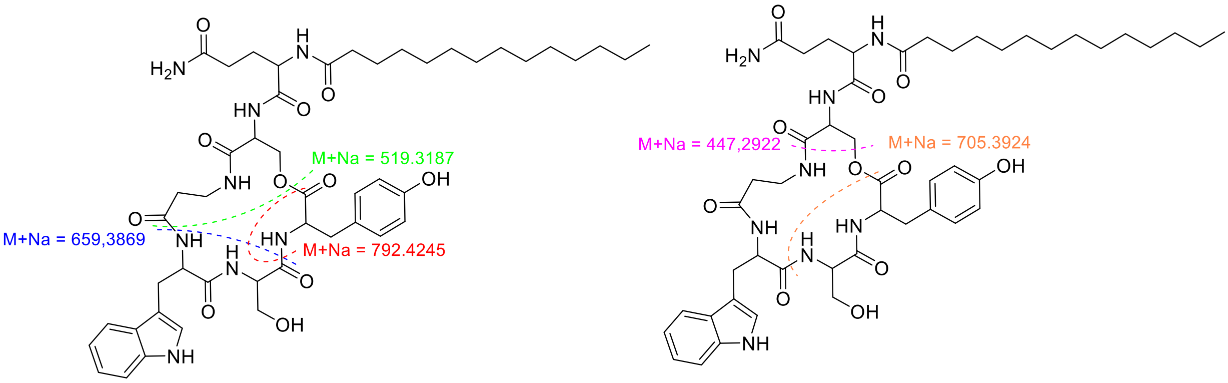

Figure S. 14: Proposed fragmentation of pleosporacin (**1**) for some of the major ion fragments observed.

IV. ^{1}H- and ^{13}C{^{1}H}-NMR Spectra

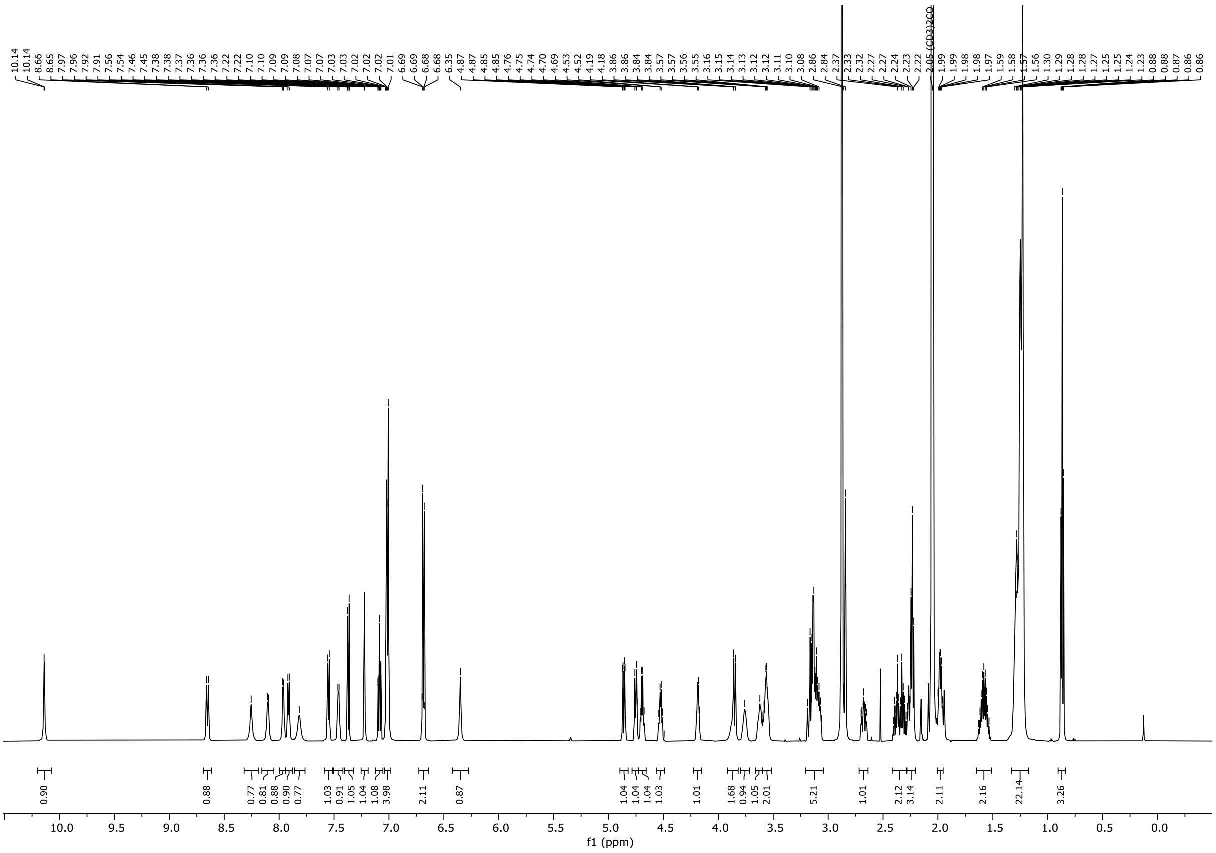

Spectrum S. 1: ^{1}H-NMR spectrum (Acetone-d$_6$, 600 MHz, 294 K) of pleosporacin (**1**).

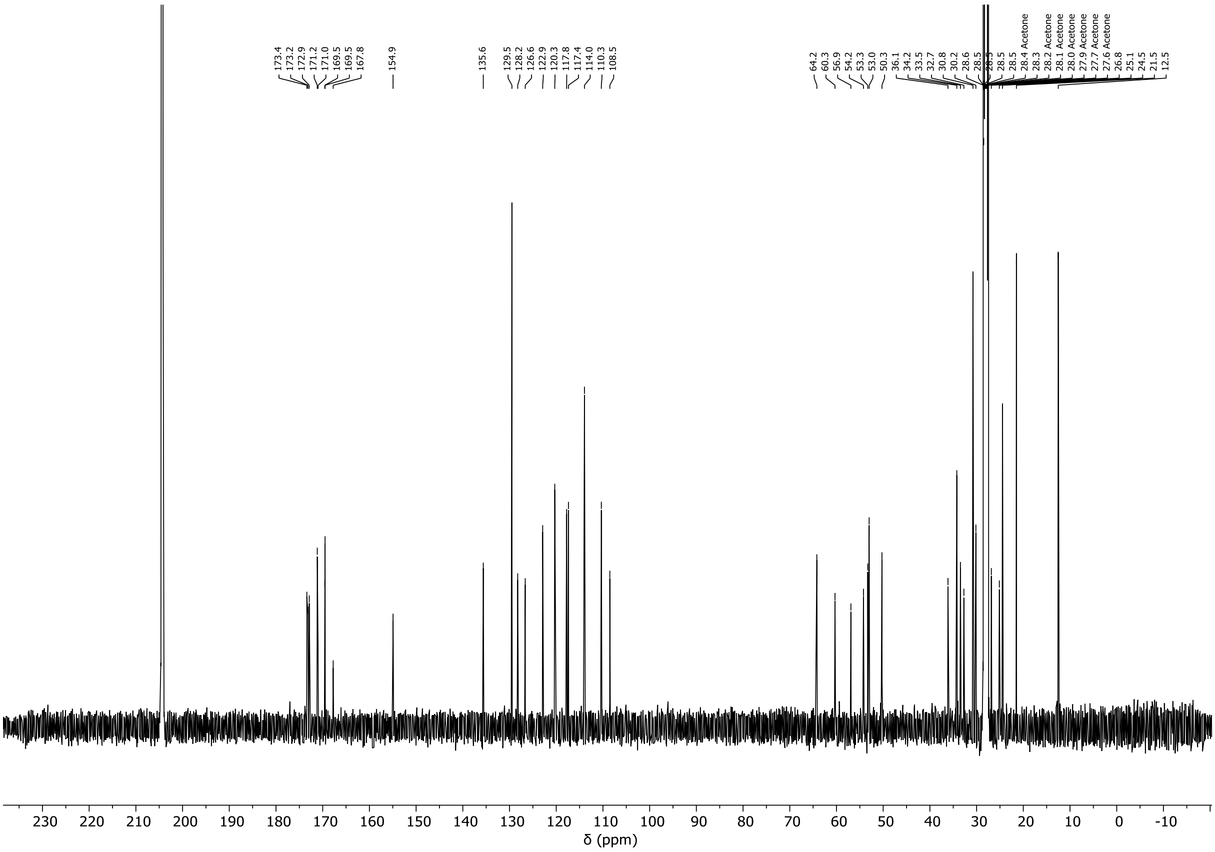

Spectrum S. 2: ^{13}C{^{1}H}-NMR spectrum (Acetone-d$_6$, 151 MHz, 294 K) of pleosporacin (**1**).

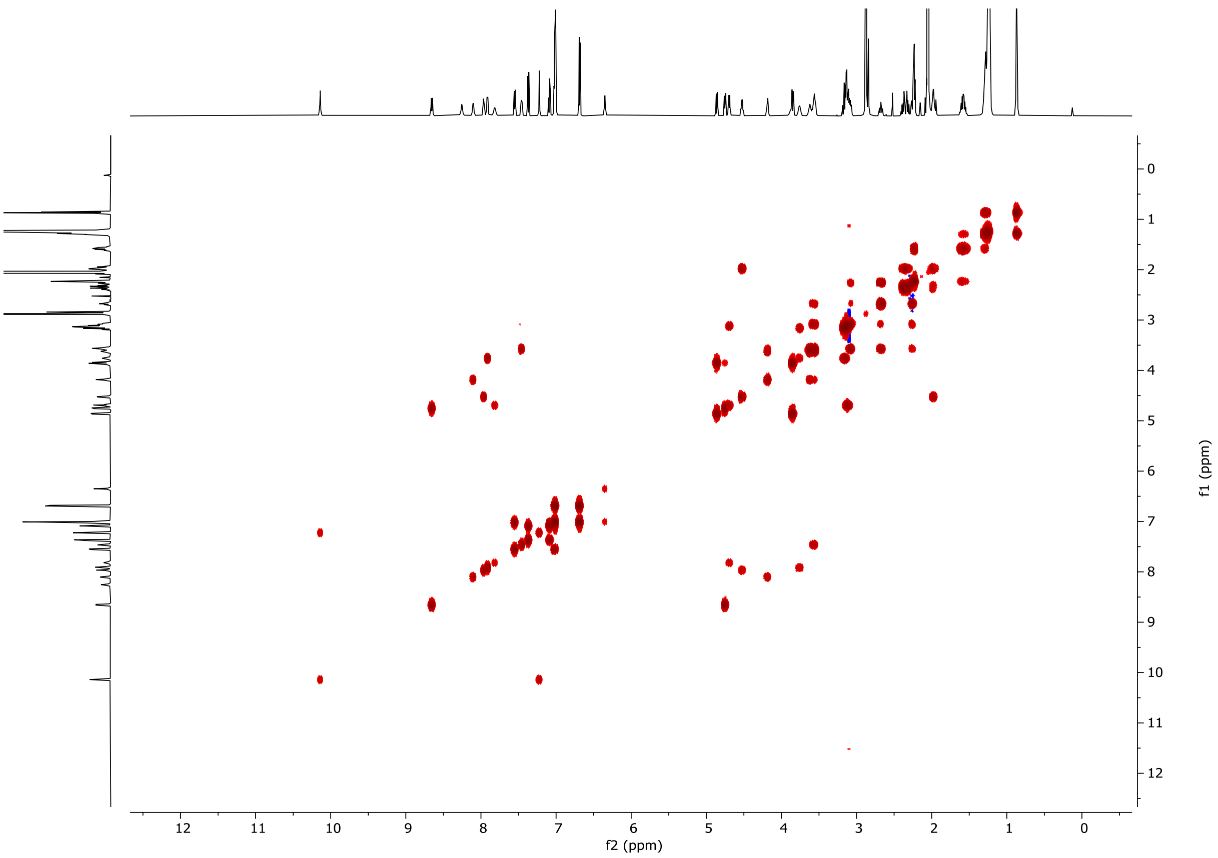

Spectrum S. 3: ^{1}H-^{1}H-COSY (Acetone-d$_6$, 600 MHz, 294 K) of pleosporacin (**1**).

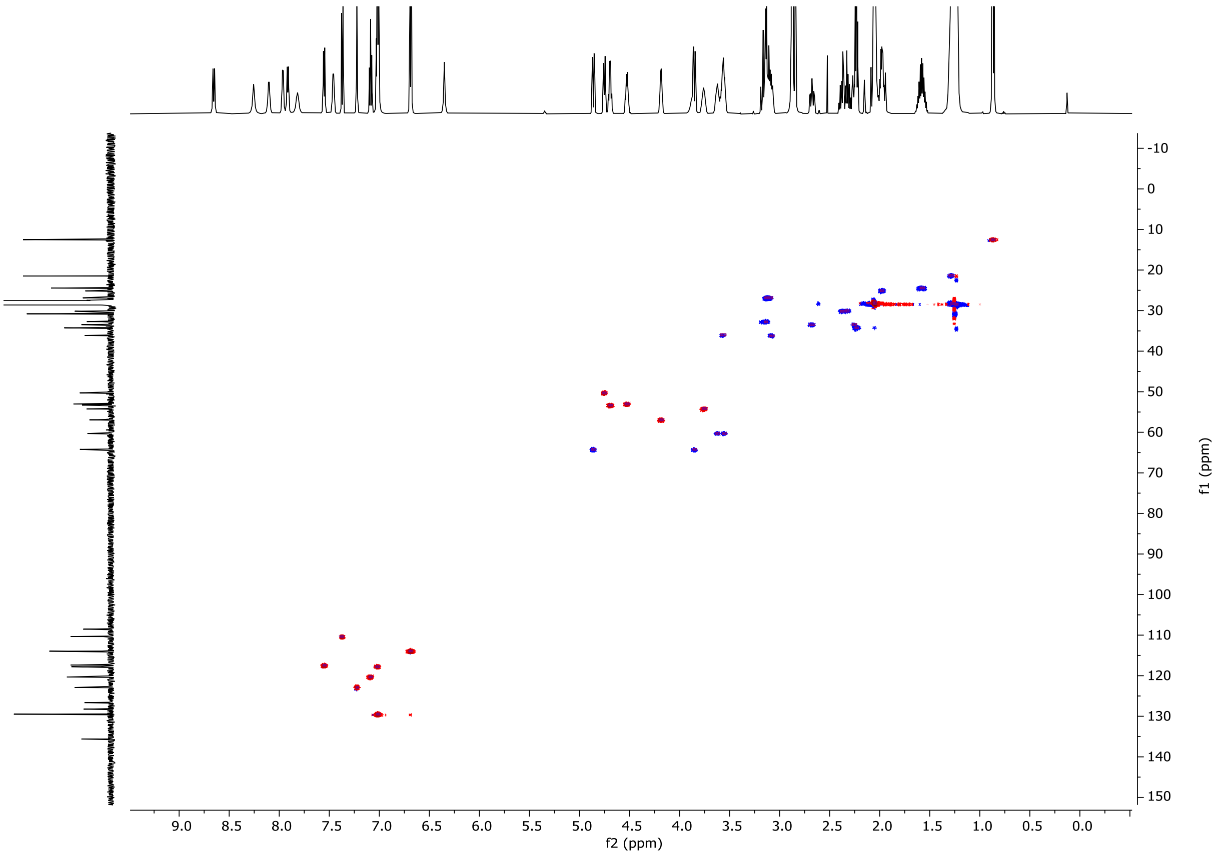

Spectrum S. 4: ^{1}H-$^{13}C\{^{1}H\}$-HSQC (Acetone-d$_6$, 600 MHz, 294 K) of pleosporacin (**1**).

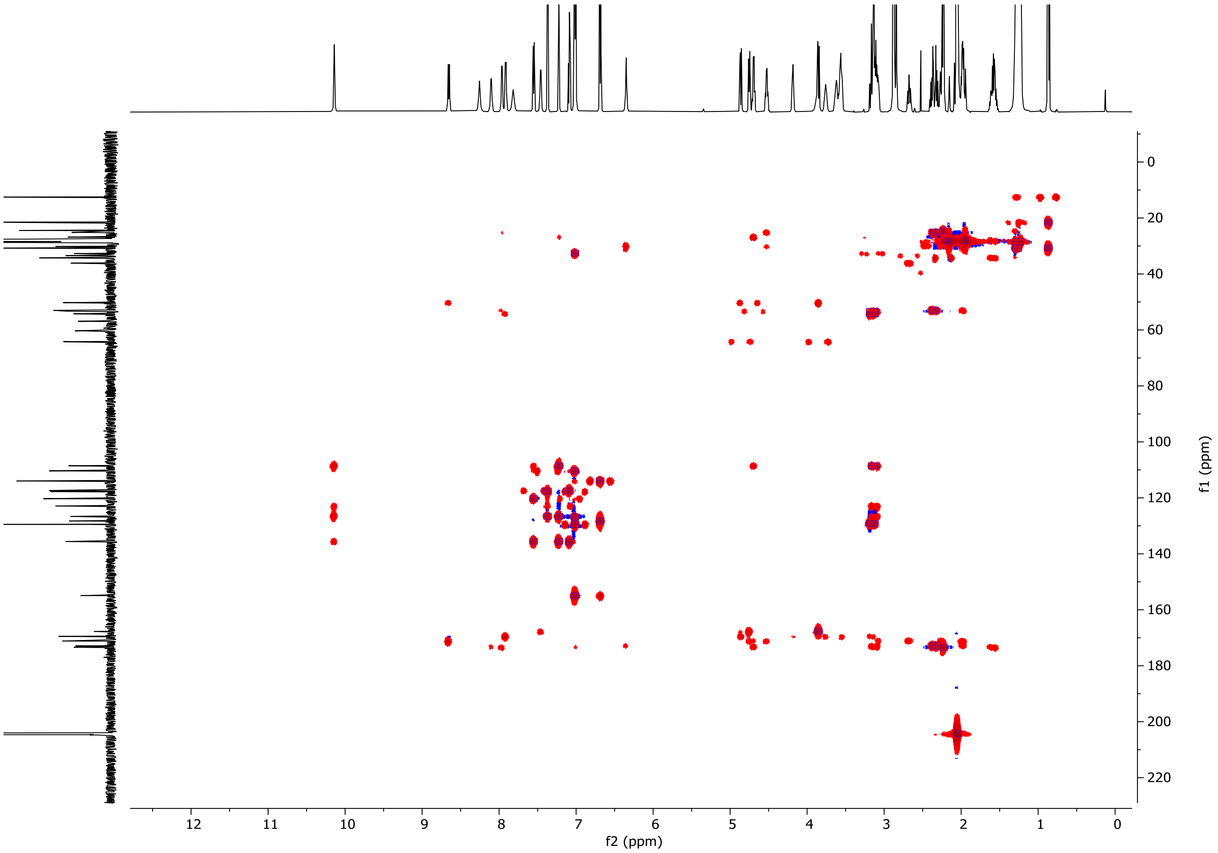

Spectrum S. 5: ^{1}H-$^{13}C\{^{1}H\}$-HMBC (Acetone-d_6, 600 MHz, 294 K) of pleosporacin (**1**).

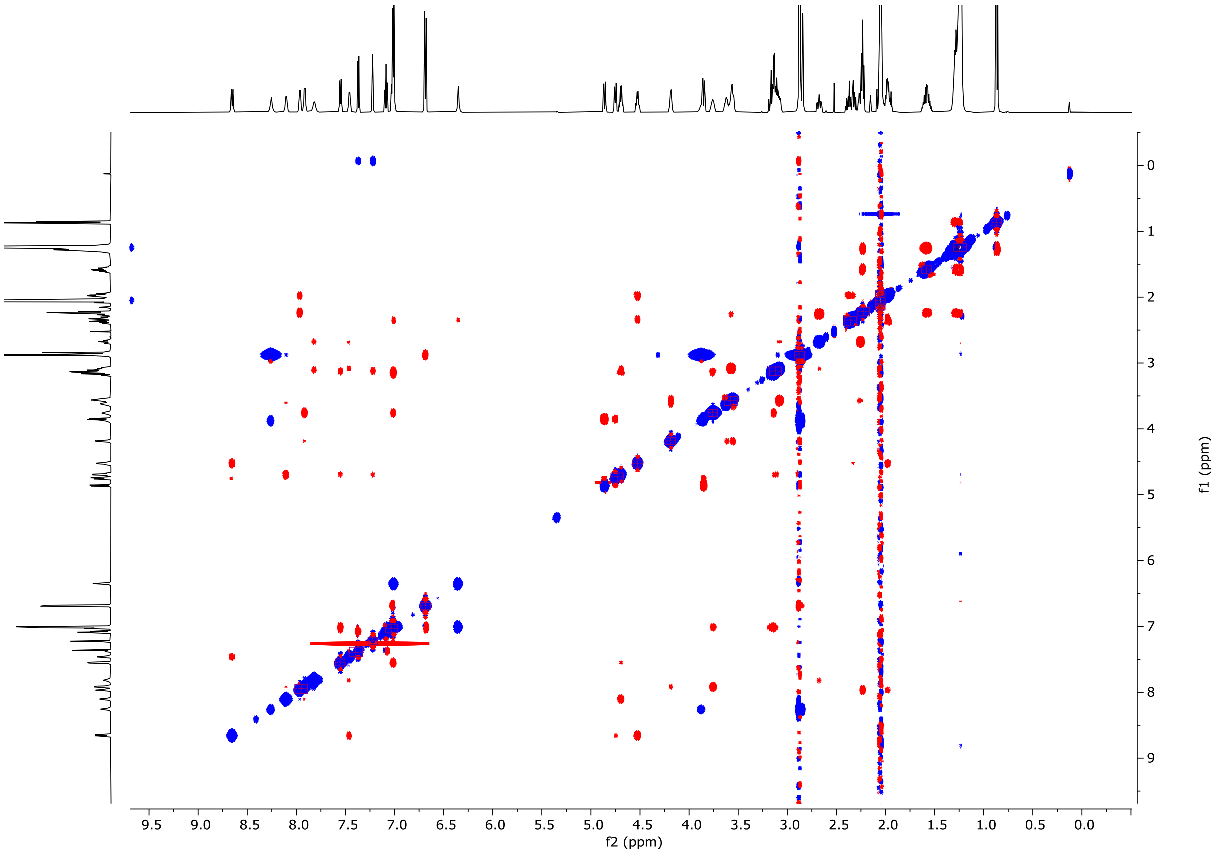

Spectrum S. 6: ^{1}H-^{1}H-NOESY (Acetone-d_6, 600 MHz, 294 K) of pleosporacin (**1**).

V. References

[1] C. Wieder, M. Künzer, R. Wiechert, K. Seipp, K. Andresen, P. Stark, A. Schüffler, T. Opatz, E. Thines, *Org. Lett.* **2025**.

[2] S. Andrews, "FastQC: a quality control tool for high throughput sequence data", to be found under https://www.bioinformatics.babraham.ac.uk/projects/fastqc/, **2010**.

[3] A. Bankevich, S. Nurk, D. Antipov, A. A. Gurevich, M. Dvorkin, A. S. Kulikov, V. M. Lesin, S. I. Nikolenko, S. Pham, A. D. Prjibelski et al., *J. Comput. Biol.* **2012**, *19*, 455–477.

[4] A. Gurevich, V. Saveliev, N. Vyahhi, G. Tesler, *Bioinformatics (Oxford, England)* **2013**, *29*, 1072–1075.

[5] H. Thorvaldsdóttir, J. T. Robinson, J. P. Mesirov, *Briefings Bioinf.* **2013**, *14*, 178–192.

[6] K. Blin, S. Shaw, H. E. Augustijn, Z. L. Reitz, F. Biermann, M. Alanjary, A. Fetter, B. R. Terlouw, W. W. Metcalf, E. J. N. Helfrich et al., *Nucleic Acids Res.* **2023**, *51*, W46-W50.

[7] J. Tang, Y. Matsuda, *Nat. Commun.* **2024**, *15*, 4312.

[8] G. R. Fulmer, A. J. M. Miller, N. H. Sherden, H. E. Gottlieb, A. Nudelman, B. M. Stoltz, J. E. Bercaw, K. I. Goldberg, *Organometallics* **2010**, *29*, 2176–2179.

[9] G. Lippke, H. Thaler, *Starch - Starke* **1970**, *22*, 344–351.

[10] H. Büttner, S. J. Pidot, K. Scherlach, C. Hertweck, *Chem. Sci.* **2022**, *14*, 103–112.

[11] K. Fujii, Y. Yahashi, T. Nakano, S. Imanishi, S. F. Baldia, K. Harada, *Tetrahedron* **2002**, *58*, 6873–6879.

[12] a) J. E. Hochlowski, P. Hill, D. N. Whittern, M. H. Scherr, R. R. Rasmussen, S. A. Dorwin, J. B. McAlpine, *J. Antibiot.* **1994**, *47*, 528–535; b) S. E. Helaly, S. Ashrafi, R. B. Teponno, S. Bernecker, A. A. Dababat, W. Maier, M. Stadler, *J. Nat. Prod.* **2018**, *81*, 2228–2234; c) T. Bunyapaiboonsri, S. Yoiprommarat, R. Suntivich, S. Preedanon, S. Komwijit, T. Teerawatananond, J. Sakayaroj, *Tetrahedron* **2020**, *76*, 131497; d) F. J. Ortíz-López, M. C. Monteiro, V. González-Menéndez, J. R. Tormo, O. Genilloud, G. F. Bills, F. Vicente, C. Zhang, T. Roemer, S. B. Singh et al., *J. Nat. Prod.* **2015**, *78*, 468–475;
